# Explainable unsupervised anomaly detection for healthcare insurance data

**DOI:** 10.1186/s12911-024-02823-6

**Published:** 2025-01-09

**Authors:** Hannes De Meulemeester, Frank De Smet, Johan van Dorst, Elise Derroitte, Bart De Moor

**Affiliations:** 1 Department of Electrical Engineering, ESAT-STADIUS, KU Leuven, Kasteelpark Arenberg 10, B-3001 Leuven, Belgium; 2 Christian Health Insurance Fund, 1031 Brussels, Belgium; 3 Department of Public Health and Primary Care, Environment and Health, KU Leuven, 3000 Leuven, Belgium

**Keywords:** Health insurance, Anomaly detection, Unsupervised machine learning

## Abstract

**Background:**

Waste and fraud are important problems for health insurers to deal with. With the advent of big data, these insurers are looking more and more towards data mining and machine learning methods to help in detecting waste and fraud. However, labeled data is costly and difficult to acquire as it requires expert investigators and known care providers with atypical behavior.

**Methods:**

In this work we show how recent advances in machine learning can be used to set up a workflow that can aid investigators in discovering practitioners or groups of practitioners with unusual resource use in order to more efficiently combat waste and fraud. We combine three different techniques, which have not been used in the context of healthcare insurance anomaly detection: categorical embeddings to deal with high-cardinality categorical variables, state-of-the-art unsupervised anomaly detection techniques to detect anomalies and Shapley additive explanations (SHAP) to explain the model output.

**Results:**

The method has been evaluated on providers with a known anomalous profile and with the help of experts of the largest health insurance fund in Belgium. The quantitative experiments show that categorical embeddings offer a significant improvement compared to standard methods and that the state-of-the-art unsupervised anomaly detection techniques generally show an improvement over traditional methods. In a practical setting, the proposed workflow with SHAP was able to detect a previously unknown, anomalous trend among general practitioners.

**Conclusions:**

The proposed workflow is able to detect known care providers with atypical behaviour and helps expert investigators in making informed decisions concerning possible fraud or overconsumption in the health insurance field.

**Supplementary Information:**

The online version contains supplementary material available at 10.1186/s12911-024-02823-6.

## Background

Healthcare and, in turn, health insurance providers are a vital pillar of a modern welfare state. However, healthcare comes at an ever increasing cost. The European Union currently spends approximately 1.5 trillion euro yearly on healthcare which, on average, corresponds to 10.9% of GDP per country in 2020 [1]. In the United States, healthcare spending reached 4.3 trillion dollars [2] which is 18.3% of its GDP. The increased standard of care that is present in modern society means having access to the latest technological advances in medicine and care, which come at a premium [3].

It is no wonder that all stakeholders involved are looking to find areas of improvement such that healthcare spending can be decreased without compromising on quality of care. One of such areas is the detection of waste and fraud. In general, waste or fraud are almost never considered on its own, but as part of a greater whole containing waste, errors, abuse, fraud and corruption [4]. The main difference between these types of infringements is the intention and degree of severity. Only in the case of abuse, fraud and corruption can it be said that there is an intent to do wrong by finding loopholes or stretching a rule (abuse) or by actively and intentionally breaking the law (fraud and corruption). In the case of errors, a rule is unintentionally broken. Similar to this is waste or overconsumption, which includes care that is unnecessary or needlessly expensive. When simply looking at the billing data, as available to healthcare insurers, it can be difficult to differentiate between these types of infringements and care providers with valid resource use. Therefore, it is common to either group the categories together and look for general anomalies. Alternatively, specific, thematic, investigations can be performed to investigate known types of fraud.

In this work, we focus on the perspective of health insurers. Health insurance funds in Belgium are co-managers of the health insurance system and have a vested interest in managing this system as optimal as possible. Additionally, these health insurers are the closest to the source as they are the first to receive detailed billing data, making them the first line of defense.

However, waste and fraud are one of the most difficult problems for health insurers to deal with. Therefore, they are looking for new ways to help them with this task. With the advent of big data, these insurers are looking more and more towards data mining and machine learning methods to help in detecting anomalies in resource use data. Investigating waste or fraud in healthcare practitioners in principle requires a business specialist to go over the resource use records of all the care providers and to subsequently interview the practitioners with a potential anomalous profile. Such manual investigations are costly, inefficient and require a lot of time without tools to guide the investigator. Such tools can help by pre-selecting the care providers with a profile that could have a higher risk of inappropriate resource use. Manual investigations are also required to acquire labeled data if one wants to apply supervised machine learning techniques. This need for expert investigators to label the training data makes supervised methods more difficult. Additionally, supervised problems are limited in that only previously known types of waste and fraud are considered. As a result, the labeled data can contain additional false negatives with respect to inappropriate resource use in general. Finally, it is usually desirable to be able to identify new types of anomalies that warrant further investigation.

This research has been performed at the Christian Health Insurance Fund (CM) [5]. The CM, which has over 4.5 million members, is the largest Belgian mutual health insurer and currently processes over 400 million expenditure records every year.

### Contribution

This work shows how a number of recent advances in machine learning can be used to improve anomaly detection on health insurance resource use data and to help healthcare experts make informed decisions about possible fraud or overconsumption in the field. We combine three different state-of-the-art techniques, which have not been used in the context of healthcare insurance anomaly detection previously, to create a novel workflow. Categorical embeddings [6] are employed to deal with the large number of categorical features and values. These embeddings allow for a more efficient representation of the data, which in turn speeds up machine learning methods and significantly increases their performance. A large variety of unsupervised anomaly detection techniques, each with their own unique advantages, are used during the experiments. We consider both classic methods, which have been a staple in the anomaly detection field, and novel methods, which have not been employed in the context of health insurance data. Finally, we illustrate how Shapley additive explanations (SHAP) [7] can be used to help experts identify anomalies and novel patterns in the data.

### Related work

Advanced statistical, data mining and machine learning techniques are a mainstay for fraud detection in many different areas [8, 9]. However, healthcare insurers are slower to implement these advanced types of techniques due to complex regulations, large decentralized data systems and high costs of commercial fraud detection packages [10] with questionable or at least unproven efficacy. Further, preparing the data and labeled data for machine learning is costly and manually intensive. Finally, investigating anomalies and subsequently, if necessary, deal with care providers with inappropriate resource use is a difficult and long process. Notwithstanding the slow integration, there has been some research into the field of unsupervised fraud detection in healthcare.

Chandola et al. [11] showed how text mining, social network analysis and temporal analysis can be applied to big healthcare data. For text mining, they performed topic modeling using latent Dirichlet allocation and determined that certain diagnosis topics were more likely to be related to fraudulent claims. Concerning social network analysis, graphs were created with the providers as nodes. They found that another fraudulent provider was present in 40% of the cases within 2-hops from a known fraudulent provider. Their temporal analysis showed that anomalies could be detected using temporal features. They also showed that features of a temporal nature are useful as input to supervised models such as logistic regression classifiers.

Thornton et al. [12] used k-means clustering, using custom metrics to perform anomaly analysis. The metrics were developed in cooperation with experts and incorporated features such as visit length, patient retention, referral rate and visit frequency. The domain knowledge used is common in their research and they conclude that this expertise is vital in order to develop healthcare fraud detection techniques [13].

Van Capelleveen et al. [14] performed experiments on dentist Medicaid data and showed that previously unknown patterns can be detected using simple unsupervised methods such as linear models and k-means clustering. The authors worked closely together with field experts and concluded that their methods could be beneficial for a decision support.

Bauder et al. [15] show that it is possible to classify healthcare professionals into their respective fields based on billing data. The practitioners for which the models have difficulty classifying them are flagged as outlying. The authors also developed a novel univariate probabilistic programming anomaly detection method which provided interpretability on the feature level [16]. The method was later extended with a MARS regression model [17, 18]. Bauder et al. additionally employed traditional unsupervised anomaly detection models where it was shown that Local Outlier Factor [19] performed the best [20].

From the existing research, it can be seen that machine learning and data mining techniques can be beneficial when used in the field of unsupervised healthcare insurance anomaly detection. However, limited research has been performed in cooperation with European health care insurers and fewer still has been performed with the latest anomaly detection and explainability techniques.

## Methods

**Fig. 1:**
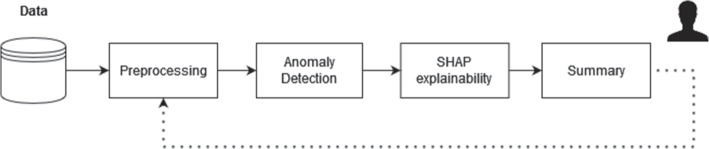
The general overview of the workflow to help expert investigators in detecting anomalous behavior and to pre-select care providers with a profile that could have a higher risk of inappropriate resource use. The health insurance data is first filtered and preprocessed to obtain a single, rich, feature vector for each practitioner. Next, anomaly detection is performed by a number of traditional and state-of-the-art anomaly detection techniques. The model output, for a sample to be investigated, is explained by using SHAP. Finally, the results are summarized in the form of visualisations and summary statistics to the expert investigator

In Fig. 1 a general overview of the workflow can be found. First, the data is preprocessed such that a suitable data set is created for the machine learning models. Next, a wide variety of unsupervised anomaly detection techniques is employed to detect and rank the actual anomalous profiles. For the anomalous samples that have been detected, SHAP is employed such that for each practitioner the feature attribution, or importance, scores are calculated. Finally, the results are summarized and visualized to help the expert investigators in interpreting the results and guiding the investigation by helping the experts to efficiently select the care provider profiles that might warrant further investigation. When employing the workflow, it was found useful to have an expert in the loop at all times such that the process can be further optimized. For example, it might turn out that a certain variable, that was included in the preprocessing, is not useful to determine anomalies or has an unwanted impact on the anomaly detection methods.

### Preprocessing

**Fig. 2:**
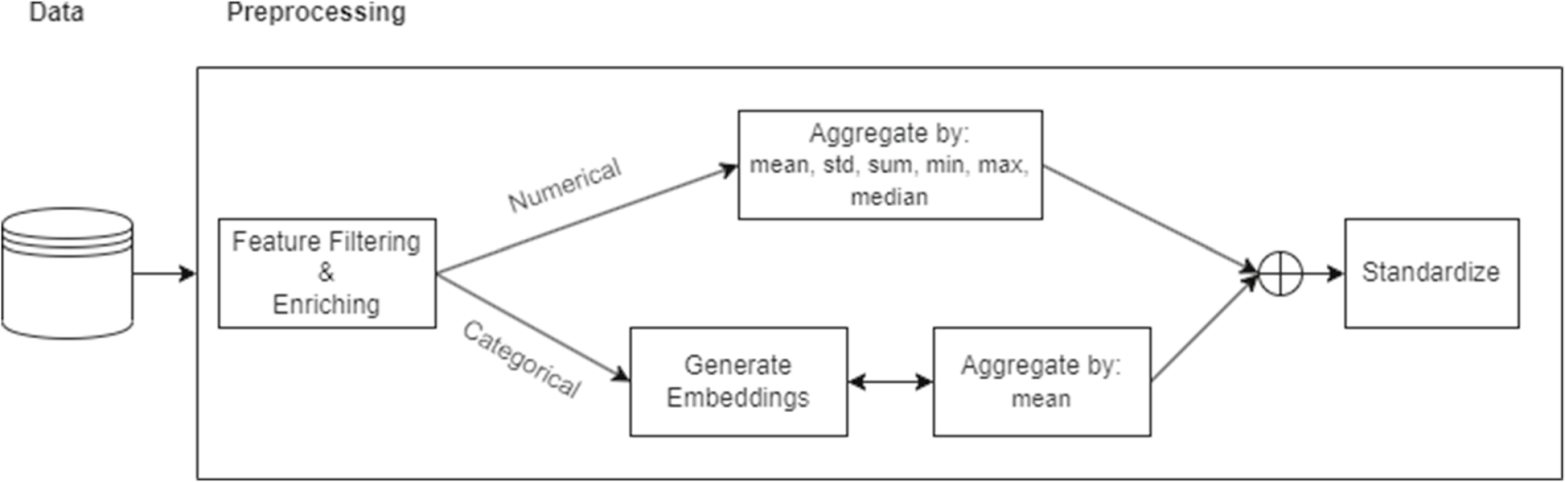
The preprocessing performed before performing anomaly detection. In close cooperation with experts, the features are filtered and enriched (e.g. using expert knowledge) such that a suitable set of the data and features are extracted that are useful in determining atypical behavior. Numerical features are aggregated per practitioner by their mean, standard deviation, sum, minimum, maximum and mean statistics. The categorical features are embedded using a categorical embedding method and aggregated using the mean. The data is then recombined and standardized by subtracting the mean and dividing by the standard deviation

Figure 2 shows a schematic overview of the preprocessing steps performed before employing the actual anomaly detection techniques. Resident experts were involved in the entire process, including the preprocessing, in order to identify useful features and interpret the data correctly. This allowed us to reduce the number of available features from hundreds to 66 different features that were identified to be potentially useful in characterizing anomalous behavior.

Typically, tabular data contains both real and categorical data. Most machine learning methods however cannot by default be applied to categorical features. Currently, the standard is to use one-hot encoding, also known as dummy encoding [21], for categorical data. For a single categorical variable, such encoding is performed as follows. Consider a categorical variable *C* with *n* unique categories $\{c_1, c_2\dots ,c_n\}$. Then a category $c_i$ is transformed into a vector $x \in \mathbb {R}^n$ where each element is defined by Eq. 1.

1 $$\begin{aligned} x_i= \left\{ \begin{array}{ll} 1 & \text{ if } C = c_i \\ 0 & \text{ if } C \ne c_i \end{array}\right. \end{aligned}$$

However, for categorical features with a high cardinality this results in long feature vectors which greatly increases the dimensionality of the data set. Additionally, not all machine learning methods are able to efficiently handle high-dimensional inputs which can be detrimental to their performance. A better alternative is to transform categorical features by using categorical embeddings [6]. These embeddings have been shown to extract useful information, such as relations between the categorical variables, and result in a much smaller feature size. It was decided to use Latent Semantic Analysis (LSA) [22] as the embedding method as this was shown to be the most consistent [6].

**Fig. 3:**
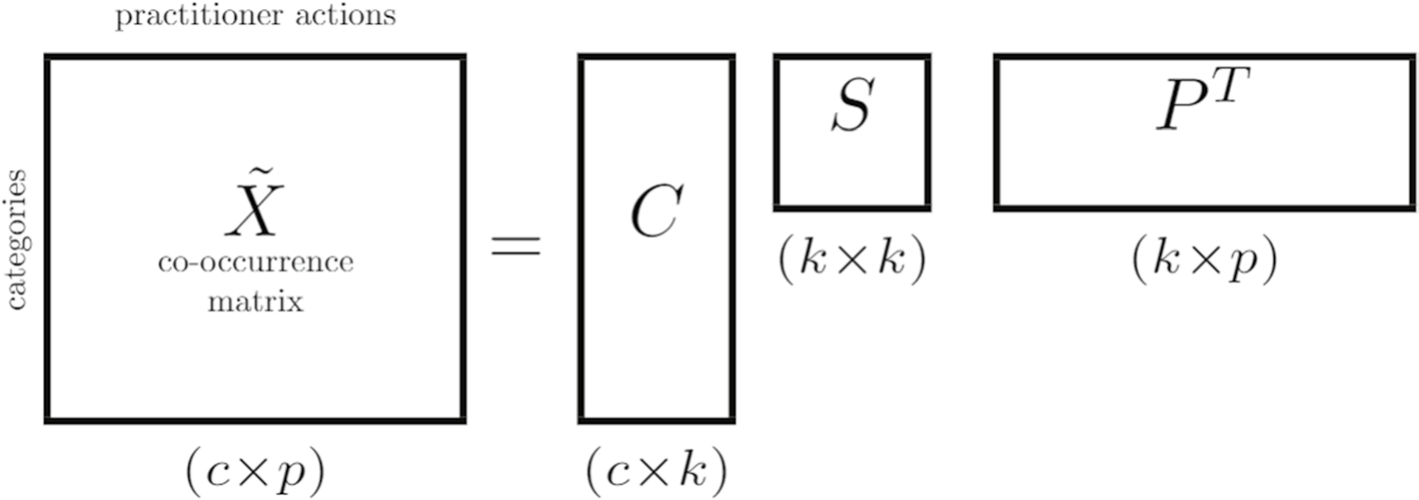
The Singular Value Decomposition step of LSA applied to categorical variables. The co-occurrence matrix, relates the categories with the data entries (the practitioner actions), gets decomposed into a rank-*k* approximation of the category space *C*, the practitioner actions space *P* and weights *S* which relates the two

LSA originates from the information retrieval field where it is used to create word and document vector representations. For categorical variables, it was shown that a similar process can be used to create vector representations of the categorical values. First, a co-occurrence matrix *X* is constructed with as rows all possible categories (from all categorical variables) and as columns all actions (of all practitioners). Then, a binary matrix is constructed such that each entry of the matrix indicates whether a specific categorical value is present in a practitioner action or not. At this point, each categorical value is represented in *X* by a large binary vector which could be used for machine learning tasks. However, this vector representation has as dimensionality *p*, the number of practitioner actions (rows) in the data set, which is often too large for any practical use. Therefore, in LSA, a rank-*k* approximation is made of *X* by means of the Singular Value Decomposition (SVD) as in Fig. 3. This approximation extracts the most important relations from the co-occurrence matrix while removing redundancy and noise. The categorical embeddings for all categories are obtained by considering only the category part of the decomposition: $XP = CS \in \mathbb {R}^{c\times k}$ where each row corresponds with the embedding of a category with dimension *k*. Finally, the resulting embeddings are l2-normalized. This normalization is typical in information retrieval as it allows, for example, to easily compare two vectors by using the cosine distance by a dot product [23].

The embedding size *k* is a tuning parameter as the optimal value depends on the problem. Generally, a trade-off is made between the size and expressivity of the vectors. Larger embeddings can capture more information, up to a point, but make the learning process slower due to the larger dimensionality. In natural language processing, large embedding sizes (e.g. $> 100$) are usually preferred [24] while for categorical embeddings, smaller sizes already achieve a significant performance increase [6].

For the embedding size, it was decided to use 3 dimensions as this showed to provide a good performance improvement during our experiments while keeping the overall dimensionality manageable. Increasing the dimensionality offered little to no performance improvement while only increasing the computational complexity. The process described above creates an embedding, in our case a 3-dimensional vector, for each categorical value possible. Each value in the original data set is then replaced (embedded) by the corresponding embedding. After embedding the categorical features, the data set contains only real-valued features.

Healthcare insurance data is time series data where each sample is an action for a particular practitioner or institution. Because of the different possible actions, varying lengths and the scale of the data, the time series are typically aggregated into a single vector per practitioner. The aggregation follows previous work [20, 25] such that all numerical features are aggregated by their sum, mean, median, standard deviation, minimum and maximum statistics. For the embedded data, the features are aggregated using the mean. Finally, the resulting feature columns are standardized before training by, for each feature, subtracting the mean and dividing by the variance.

### Models

The experiments are performed using a wide variety of anomaly detection methods to investigate which types of algorithms are most suitable. This section gives a short overview of these methods.

**Nearest neighbor** methods are commonly used methods due to their simplicity and ease to explain. These methods take into account the *k* nearest neighbors of a data point to calculate their outlyingness score. In the simplest case, for the K Nearest Neighbors algorithm, the distance between a sample and its *k* nearest neighbors can be calculated and averaged. This average distance can then be used to rank the samples such that anomalies get a higher outlyingness score because they are furthest from their neighbors. A disadvantage of these methods is that they are generally less effective for high-dimensional problems as the effect of any single feature becomes less important when calculating the distance between two samples. K Nearest Neighbors (KNN) [26] and Local Outlier Factor (LOF) [19] have been successfully used on Medicare data [20] where these methods were the best performers. Additionally, we consider Angle-based Outlier Detection (ABOD) [27] and Subspace Outlier Detection (SOD) [28] which have been shown to work better on high-dimensional data. The final nearest neighbor method that is employed is Learnable Unified Neighborhood-based Anomaly Ranking (LUNAR) [29], a state-of-the-art local anomaly detection technique. This model combines the nearest neighbor approach with graph neural networks.

**Isolation** based methods take the entire data set into account and attempt to partition the data into regions such that small outlying groups are easily isolated from the rest of the data. These methods generally employ decision trees, which makes them explainable and fast because they are easily parallizable. Furthermore, the interpretability of decision tree methods lends itself well to create trustworthy AI systems [30]. Isolation Forest (IForest or IF) [31] use an ensemble of binary decision trees constructed from a random sample of the data. The anomalies are isolated by randomly splitting the data at each level based on a random feature. An outlyingness score is calculated based on the length of the path down the decision trees of a sample. Unsupervised Random Forest (URF) [32] uses the standard random forest in an unsupervised setting by introducing synthetic data. The proximity matrix provided by the random forest algorithm is then used to calculate an anomaly score. A more recent method in this category is called Isolation-based Nearest-Neighbor Ensembles (INNE) [33]. It was shown that this method outperforms Isolation Forest models when it comes to detecting local anomalies and high-dimensional data with many irrelevant features.

**One-class** methods attempt to learn the single class that is represented by the training data. Typically, such methods attempt to find a suitable trade-off between a hypersphere that encompasses as much of the training data as possible while at the same time keeping the hypersphere small. The most well-known of these methods is the One-Class Support Vector Machine (OCSVM) [34]. Deep Support Vector Data Description (DeepSVDD) [35] can be seen as an extension to the OCSVM where the encompassing hypersphere is learned together with non-linear feature representations by using a deep neural network.

**Distribution estimation** methods attempt to learn the data distribution, or an approximation thereof, which can then be used to identify anomalies. A Variational Autoencoder (VAE) is a probabilistic generative model in the form of an autoencoder. The reconstruction probability of a trained VAE can be used as an anomaly score. Lightweight On-line Detector of Anomalies (LODA) [36] is a method where the data set is projected on a number of random vectors of which an ensemble of one-dimensional histograms is then created. The method takes inspiration from ensemble and boosting methods and shows that a collection of weak models can achieve state-of-the-art results while being one of the fastest anomaly detection methods. Copula-Based Outlier Detection (COPOD) [37] and Empirical-Cumulative-distribution-based Outlier Detection (ECOD) [38] are two recent parameter-free methods. The methods use different ways to inspect, for each dimension, the tails of their univariate empirical cumulative distribution function and aggregate this information into an anomaly score.

### Explainability

Machine learning approaches have been slow to be adapted for use in the healthcare insurance field. One important reason for this is that it can be difficult to interpret and explain the model output. Understanding why a machine learning model makes a prediction is a requirement if it is to be used by experts in order to make a decision concerning the presence of fraud or overconsumption.

**Fig. 4:**
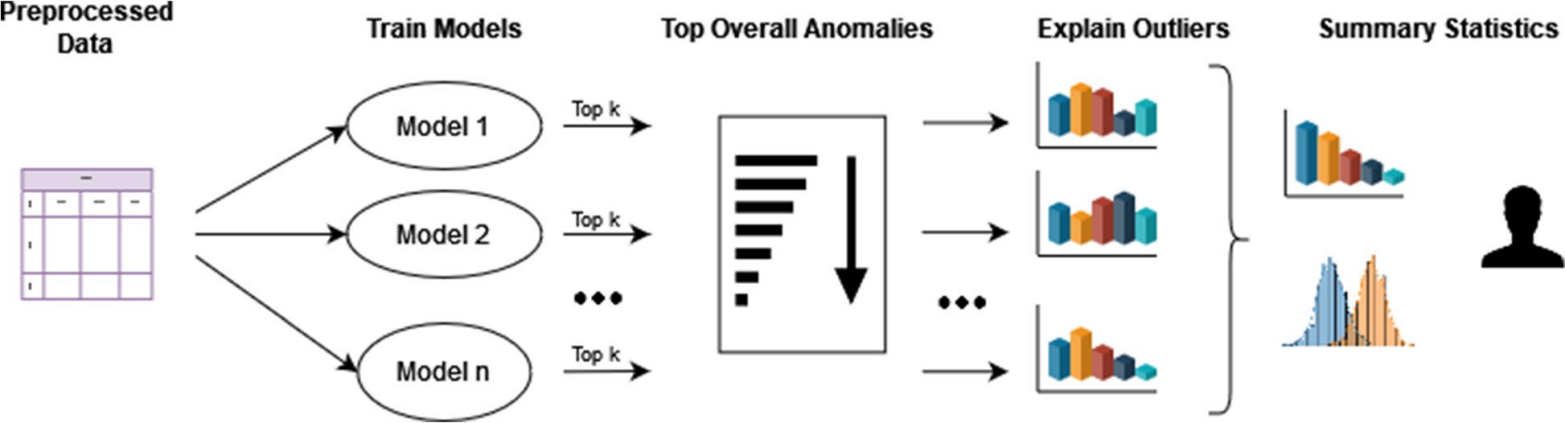
The process for explainable anomaly detection. Preprocessed data is provided to *n* anomaly detection models. The top *k* anomalies for each models are explained using SHAP. Next, the anomalies are ranked based on how many times they are detected by the anomaly detection methods. finally, The explanations in combination with standard statistics are summarized and provided to an expert to help them in their decision making

Recently, a model-agnostic technique, called SHAP [7], was proposed that allows any model output to be explained. The method can assign an importance value to any feature for a single prediction or a group of predictions by using Shapley values [39], originating from cooperative game theory. SHAP adds a new dimension to machine learning explainability and has been the foundation for new research directions. For example, an extension to SHAP, Asymmetric Shapley Values [40, 41], allows domain knowledge to be directly included in the explanation process. Calculating SHAP values is computationally costly except when using linear or tree-based models [7]. However, it is not necessary to calculate these values for the entire data set as only the top outlying samples are of interest. Figure 4 shows how SHAP is incorporated in the workflow. For the trained anomaly detection models, the top *k* outlying care providers are selected. Each of these samples may be considered separately and investigated individually. However, to further limit the search window, we found it informative to sort the anomalies based on the number of times it is present in the top *k* for all models. As a result, an expert is provided a list of profiles with unusual resource use where the most anomalous, over all models, are ranked higher.

**Fig. 5:**
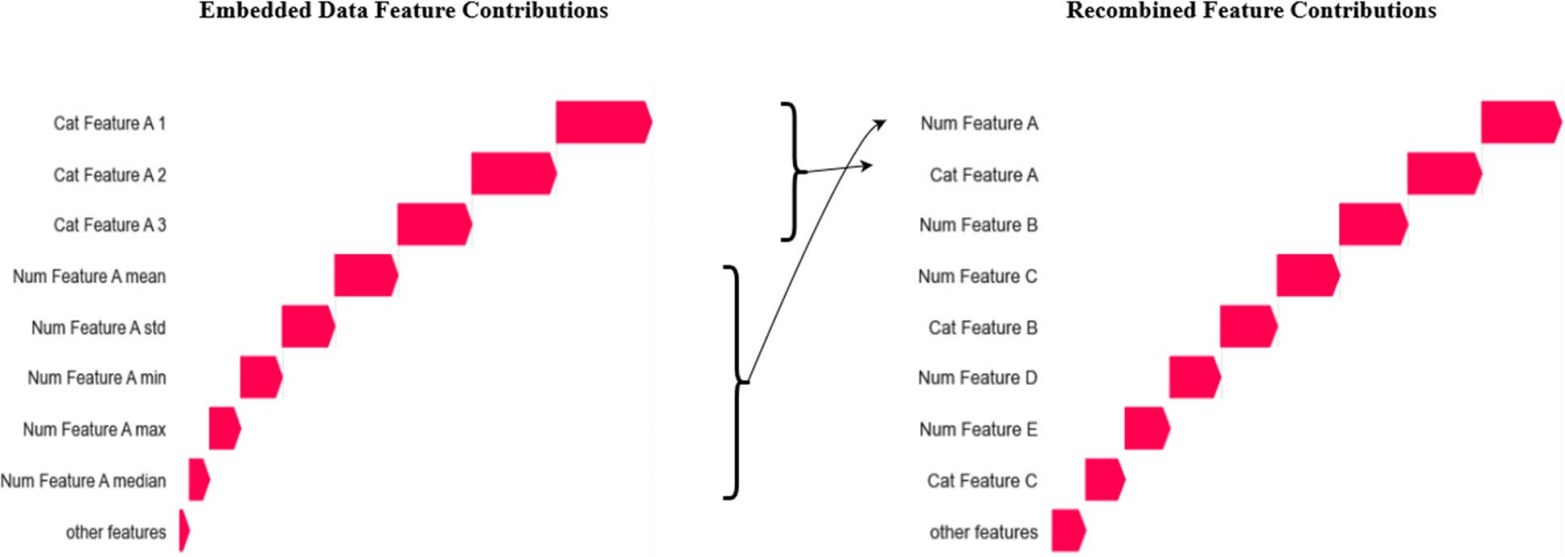
The SHAP feature contributions for the feature embeddings are summed such that the contribution of the original feature is achieved. For a numerical feature, all values are aggregated per practitioner using the mean, standard deviated, minimum, maximum and median. For a categorical variable, the values are embedded and aggregated by the mean. Both methods expand a feature into multiple dimensions which means there are as many feature contributions (importance scores) per original feature. SHAP has the advantage that these scores can be summed to achieve the overall importance score for the original feature

A large advantage of SHAP is that Shapley values are fully additive, which means that the respective SHAP values of multiple features can be added to get their corresponding total importance. This is especially useful when working with embeddings, or feature statistics, where a single feature is transformed into a new representation with a larger dimensionality. In such cases, it is often not easy to calculate an importance score for the original feature using traditional methods, whereas using SHAP we simply need to sum the scores for all dimensions. This process is illustrated in Fig. 5. The left graph shows the feature contribution of the categorical embeddings and numerical feature statistics. For both categorical and numerical features, the dimensionality of the input features is larger than that of the original features. The feature contributions of all new dimensions are summed to find the total importance of a feature with regards to predicting the anomaly score of a practitioner.

At this point, the top anomalous samples over all models are listed together with their SHAP feature contributions. The investigator is able to see why a model assigns a high anomaly score to a practitioner. This is useful as different techniques might flag a sample as outlying for different reasons. However, a single explanation for all models provides a better overview, especially when working with many different models. Therefore, all SHAP scores are rescaled and the mean over all models is calculated to provide a single plot that shows the feature contributions over all models. Finally, it was found to be useful to provide additional statistics from the original time series data. Specifically, for an outlying sample, the SHAP values of the top aggregated features are shown together with the univariate feature distributions of the time series data for the top outlying features. For the categorical features, the distribution of the practitioner is shown together with that of the population. For the numerical features, the values are compared with the average population values and standard univarate statistics such as the Z-score are shown.

One important feature that is always provided to the expert in the form of summary statistics is the nomenclature for the action performed by the institution or the practitioner. For example, in the United States, these are the HCPCS codes [42]. In Belgium, these codes are simply referred to as nomenclature codes [43].

**Fig. 6:**
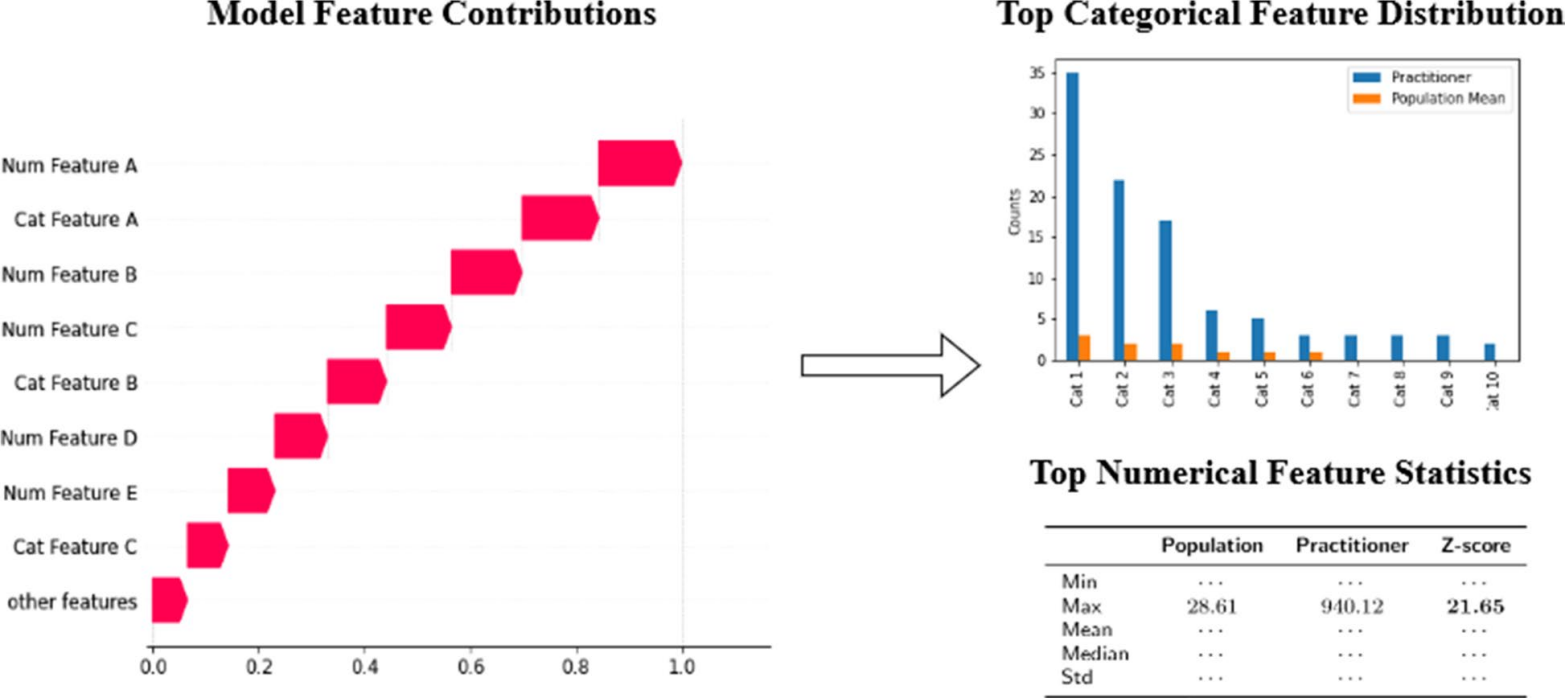
The SHAP model feature contributions, for a single sample, indicate which feature contribute most to the model output i.e. the anomaly score. The features contributing most to the sample being considered anomalous are then further investigated. For a categorical feature, the distribution of the categorical values are shown and compared to the average of all practitioners. For a numerical feature, the aggregate features (i.e. the minimum, maximum, mean, median and standard deviation) are shown, compared with the mean of the all practitioners and the Z-score is provided. This provides a clear overview for the expert investigator that shows why a practitioner’s behavior is flagged as anomalous

The final result is a clear overview where a field expert can immediately identify why a sample is flagged as outlying and whether further investigation for the sample is needed. An example of an importance plot with univariate statistics for a categorical and numerical feature is shown in Fig. 6. The top anomalous practitioners are investigated by first inspecting the overall SHAP importance plot. The expert will then investigate the summary statistics of the most important features in combination with the nomenclature codes in order to identify the nature of the anomaly. The proposed workflow filters and sorts the anomalous practitioners, reducing the search window, and provides an explanation for the detection.

## Results and discussion

### Belgian health insurance data

The CM data analyzed in this paper has only been used for research purposes, as a proof of concept, without any operational goals. All research has been performed on pseudonymized data at the CM under the supervision of the chief medical officer. No re-idenfitication of the data points has been performed at any time, regardless of whether the samples have been identified as anomalous or not.

In this section, we consider the data set of all general practitioners (GPs) in Belgium that have more than 50 entries per year in the CM database. This results in 11851 different GPs. A set of 38 general practitioners was provided who were known for their anomalous profiles due to excessive use of certain billing codes. This setting is a decidedly imbalanced task where only 0.3% of the data set are identified as known anomalies. It is important to note that there could be, and likely are, more unknown anomalies present in the data. The original data contains 12899707 individual medical acts, in the form of resource use data, of all general practitioners. Each practitioner action has 66 features of which 54 contain categorical features and 12 contain numerical features. The cardinality for the categorical features varies between 2 and 159155. After the preprocessing, described in the previous section, the data sets have the final number of features and samples as shown in Table 1.

**Table 1:** The number of samples and features of the data sets used during the experiments. The original data is aggregated and transformed into one-hot encoded and embedding data sets

Data	Samples	Features
Original	12899707	66
One-hot	11851	191707
Embeddings	11851	234

### Experiment results

The performance of the anomaly detection methods is quantified using the area under the ROC curve (AUROC). All models output a real-valued anomaly score, which is used to sort the samples with more anomalous samples being ranked higher. The ROC curve is calculated using these anomaly scores and labels indicating whether the practitioner has a known anomalous profile.

The AUROC has two advantages which makes it suitable to the discussed setting. It is immune to class imbalance, which is certainly the case, and it assigns a higher score when the outlying class is ranked higher than the other class. This second point makes it especially suitable as one goal is to investigate which anomaly detection methods can best help experts with identifying novel and outlying samples, which would be the methods that are able to rank anomalies higher.

The models used in the experiments have been trained using parameters similar to those recommended in previous work. Only minimal tuning has been performed so as not to overfit on the limited number of available labels. The hyperparameter values can be found in the supplementary material. The experiments were performed using Python where the models have been implemented using the Scikit-learn [44], PyOD [45] and Tensorflow [46] packages.

In Table 2 the results of the experiments on the transformed CM data sets can be found.

**Table 2:** Area under the ROC curve of for the one-hot and embedding encoded CM general practitioner data for the following algorithms: Local Outlier Factor (LOF), K Nearest Neighbors (KNN), Isolation Forest (IF), Unsupervised Random Forest (URF), Angle-base Outlier Detection (ABOD), Empirical-Cumulative-distribution-based Outlier Detection (ECOD), Copula-Based Outlier Detection (COPOD), Variational Autoencoder (VAE), Histogram-based Outlier Score (HBOS), Isolation-based Nearest-Neighbor Ensembles (INNE), One-Class Support Vector Machine (OCSVM), Deep Support Vector Data Description (DeepSVDD), Subspace Outlier Detection (SOD), Learnable Unified Neighborhood-based Anomaly Ranking (LUNAR) and Lightweight On-line Detector of Anomalies (LODA). For the stochastic algorithms, the performance shown is the average with standard deviation over 10 different runs. The embedding method offers a clear performance increase for all methods. VAE and LODA achieve the best performance with INNE, OCSVM and COPOD following closely behind

Model	Onehot	Embeddings
LOF	0.60	0.69
KNN	0.60	0.78
IF	$0.53~(\pm 0.04)$	$0.76~(\pm 0.03)$
URF	$0.51~(\pm 0.01)$	$0.62~(\pm 0.01)$
ABOD	0.60	0.76
ECOD	0.60	0.75
COPOD	0.60	0.81
VAE	$0.51~(\pm 0.00)$	$\varvec{0.82~(\pm 0.00)}$
HBOS	0.51	0.79
INNE	$0.52~(\pm 0.01)$	$0.80~(\pm 0.01)$
OCSVM	0.60	0.81
DeepSVDD	$0.58 ~(\pm 0.02)$	$0.61 ~(\pm 0.03)$
SOD	0.61	0.77
LUNAR	$0.77~(\pm 0.01)$	$0.78~(\pm 0.01)$
LODA	$0.56~(\pm 0.01)$	$\varvec{0.82~(\pm 0.01)}$

A clear difference can be observed between the one-hot encoded and the embedding data. For almost all methods, the embedding method significantly outperforms the current standard, one-hot encoding. Healthcare insurance data sets are rich in categorical features with often thousands of different values. One-hot encoding makes the number of features explode in size while not providing any additional useful information, which is detrimental for the performance and time complexity of most machine learning methods. The only exception to this observation is the LUNAR method. The learnability aspect of the graph neural network approach is able to overcome the limitation of standard neighbor methods where their performance tends to deteriorate for very high-dimensional data.

Overall, the best performing methods are VAE and LODA with INNE, OCSVM and COPOD following closely behind the proposed embedding method. It can be seen that the more recent and state-of-the-art anomaly detection methods generally outperform methods such as KNN and isolation forest which are traditionally used for health insurance data. The exception to this is the one-class SVM which is one of the best performing methods on the embedding data.

The experiments show that unsupervised anomaly detection methods can help to detect or prioritize the same anomalies that have been detected by experts. However, to do so effectively, special care must be taken during the preprocessing step, particularly with regards to the categorical features.

### Explainability

In this section, we show how unsupervised machine learning methods in combination with SHAP can be used to aid decision making and help experts in discovering new types of anomalies. This will be done by considering a real-life example of a novel trend that was discovered while performing the experiments.

When investigating the top anomalous profiles of general practitioners, a particular practitioner had high SHAP feature contribution scores for the features indicating the discipline, location, subcategory and the total billing amount of the medical intervention. When investigating the provided univariate feature statistics, the billing amount was significantly higher than is standard for general practitioners. The feature distribution for the discipline showed that most actions were part of the orthopedics discipline. The type of the action showed a large amount of actions related to surgeries. The location indicated that these took place at general hospitals. Finally, the distribution of nomenclature codes revealed actions such as arthroplasty of the knee or hip using a prosthesis, meniscectomy etc. As a summary, this investigation into the practitioner showed that they mainly aided in surgeries involving orthopedics in a hospital.

During further investigation, multiple practitioners were identified by the algorithm with similar behavior, indicating a trend which was previously unknown to experts at the CM. While this behavior by itself does not indicate fraud or waste, it is clearly outlying for a standard general practitioner and, as such, provides novel avenues of investigation for experts. Important for the experts was that the results of the used anomaly detection techniques were interpretable: the investigator was able to immediately identify the anomalous behavior using the combination of SHAP, simple univariate statistics and visualizations.

## Conclusion

In this work, we discussed a workflow for explainable anomaly detection in the healthcare insurance field. The workflow implements and applies a number of recent advances in machine learning: categorical embeddings, state-of-the-art anomaly detection methods and SHAP explainability, which are novel to unsupervised anomaly detection in healthcare insurance. The experiments show that unsupervised anomaly detection techniques can successfully detect and prioritize practitioners with known anomalous behavior, which were provided by experts. It was shown that the current standard of one-hot encoding categorical features is detrimental for most models due to the high cardinality of the categorical features, which are common in healthcare insurance data. Instead, we propose to use categorical embeddings, which provide a significant increase in performance.

Finally, we showed how the machine learning techniques can be used in combination with SHAP and simple summary statistics to explain the predicted anomalies and aid expert investigators. We illustrated how the approach was successfully used in the field by providing an example of a previously unknown anomalous trend for general practitioners.

## Supplementary Information

Supplementary Material 1.

## Data Availability

The data that support the findings of this study are available from the Christian Health Insurance Fund but restrictions apply to the availability of these data, which were used under license for the current study, and so are not publicly available. Data are however available from the authors upon reasonable request and with permission of the Christian Health Insurance Fund.

## Abbreviations

GDP: Gross domestic product
SHAP: Shapley Additive Explanations
CM: Christian Health Insurance Fund
ROC: Receiver Operating Characteristic
AUROC: Area Under the ROC curve
SVM: Support Vector Machine
SVD: Singular Value Decomposition
LSA: Latent Semantic Analysis
LOF: Local Outlier Factor
KNN: K Nearest Neighbors
IF: Isolation Forest
URF: Unsupervised Random Forest
ABOD: Angle-base Outlier Detection
ECOD: Empirical-Cumulative-distribution-based Outlier Detection
COPOD: Copula-Based Outlier Detection
VAE: Variational Autoencoder
HBOS: Histogram-based Outlier Score
INNE: Isolation-based Nearest-Neighbor Ensembles
OCSVM: One-Class Support Vector Machine
DeepSVDD: Deep Support Vector Data Description
SOD: Subspace Outlier Detection
LUNAR: Learnable Unified Neighborhood-based Anomaly Ranking
LODA: Lightweight On-line Detector of Anomalies
