# Supplementary material for: Explainable unsupervised anomaly detection for healthcare insurance data
Source: BMC Med Inform Decis Mak. 2025 Jan 9;25:14. doi: 10.1186/s12911-024-02823-6 (PMC11720628; doi:10.1186/s12911-024-02823-6)
Supplement: Supplementary file 1 — Supplementary Material 1. [file 12911_2024_2823_MOESM1_ESM.pdf]

Supplementary Material

Explainable Unsupervised Anomaly Detection for Healthcare Insurance Data

Hyperparameters

Table S1 The values of the hyperparameters used during the experiments. Parameters that are not mentioned use the default values of their respective implementations.

Model	Parameter	Description	Value
LOF	n_neighbors	Number of neighbors	50
	metric	The distance metric for the nearest neighbor calculation	Euclidean
KNN	n_neighbors	Number of neighbors	50
	metric	The distance metric for the nearest neighbor calculation	Euclidean
IF	n_trees	Number of trees in the ensemble	100
URF	n_trees	Number of trees in the ensemble. Uses the scikit-learn RandomForestClassifier.	100
ABOD	n_neighbors	Number of neighbors	50
HBOS	n_bins	Number of bins	50
INNE	n_estimators	Number of sets of hyperspheres in the ensemble	100
OCSVM	kernel	The kernel function	RBF
	γ	The γ parameter of the RBF kernel: $\exp\left(-\gamma \left\lVert x - x' \right\rVert^2\right)$	$\frac{1}{\text{n\_features}}$
SOD	n_neighbors	Number of neighbors	50
	ref_set	The number of neighbors in the reference set	10
LUNAR	n_neighbors	Number of neighbors	20
	model_type	Type of the neural network model. Weights indicates the model outputs weights for the nearest neighbor distances.	Weights
	negative_sampling	Type of negative samples. Mixed indicates both uniform and subspace sampling.	Mixed
LODA	n_bins	Number of histogram bins	100
	n_random_cuts	Number of random cuts	1000

Table S2 The neural network encoder (left) and decoder (right) architectures for the VAE experiments. The models have been trained using the Adam optimizer with a learning rate of 0.001.

Layer	Output	Activation
Input	219	-
Dense	128	ReLU
Dropout(0.2)	128	-
Dense	64	ReLU
Dropout(0.2)	64	-
Dense	32	ReLU
Dropout(0.2)	32	-
Dense	2	-

Layer	Output	Activation
Input	2	-
Dense	32	ReLU
Dropout(0.2)	32	-
Dense	64	ReLU
Dropout(0.2)	64	-
Dense	128	ReLU
Dropout(0.2)	128	-
Dense	219	-

Table S3 The neural network architecture for the DeepSVDD experiments. The models have been trained using the Adam optimizer with a learning rate of 0.001

Layer	Output	Activation
Input	219	-
Dense	128	ReLU
Dropout(0.2)	128	-
Dense	64	ReLU
Dropout(0.2)	64	-
Dense	32	sigmoid

Table S4 The neural network architecture for the LUNAR experiments. The models have been trained using the Adam optimizer with a learning rate of 0.001

Layer	Output	Activation
Input	20	-
Dense	256	ReLU
Dense	256	ReLU
Dense	256	ReLU
LayerNorm	256	-
Dense	20	softmax
